# Supplementary material for: Symbiotic Plant Biomass Decomposition in Fungus-Growing Termites
Source: Insects. 2019 Mar 28;10(4):87. doi: 10.3390/insects10040087 (PMC6523192; doi:10.3390/insects10040087)
Supplement: Supplementary file 1 [file insects-10-00087-s001.pdf]

**Table S1.** Studies that have reported forage substrates in fungus-growing termite species, summarized in Figure 2 of the main text. Species names are given as reported in the respective references. The references Eggleton et al. [1] and Nkunika [2] did not explain what the taxonomical annotations 'near' and '?' mean in their studies.

| Termite species | Forage substrate | Reference |
|---|---|---|
| *Macrotermes* sp. | wood and grass | [3] |
| *Macrotermes* sp. | wood, grass and leaf litter | [4] |
| *Macrotermes* sp. | dead plant material | [5] |
| *Macrotermes* sp. | dung, wood and grass litter | [6] |
| *Macrotermes* sp. | dung, wood and grass litter | [7] |
| *Macrotermes* spp.1 | grass litter | [8] |
| *Macrotermes* spp.2 | grass litter | [8] |
| *Macrotermes* spp.3 | grass litter | [8] |
| *Macrotermes* spp.4 | grass litter | [8] |
| *Macrotermes michaelseni* | grass litter, roots and fresh plants | [9] |
| *Macrotermes michaelseni* | dead roots | [10] |
| *Macrotermes michaelseni* | woody litter, elephant dung | [11] |
| *Macrotermes michaelseni* | woody litter, eucalyptus fresh and decaying leaves | [12] |
| *Macrotermes michaelseni* | standing dead grass, grass litter | [13] |
| *Macrotermes michaelseni* | dead wood | [14] |
| *Macrotermes michaelseni* | live grass | [5] |
| *Macrotermes michaelseni* | wood and grass litter | [15] |
| *Macrotermes subhyalinus* | grass litter, fresh plant material and roots (the last if litter is in short supply) | [16] |
| *Macrotermes subhyalinus* | leaves and dead wood fragments from trees or logs | [17] |
| *Macrotermes subhyalinus* | log and twigs | [18] |
| *Macrotermes subhyalinus* | dung, wood and grass litter | [15] |
| *Macrotermes subhyalinus* | millet canes, dung, wood litter | [19] |
| *Macrotermes bellicosus* | dead wood fragments from trees or logs | [17] |
| *Macrotermes bellicosus* | log and twigs | [18] |
| *Macrotermes bellicosus* | grass litter | [8] |
| *Macrotermes bellicosus* | live wood | [20] |
| *Macrotermes bellicosus* | dead grass | [21] |
| *Macrotermes herus* | grass litter | [8] |
| *Macrotermes gilvus* | fresh leaves and grass | [22] |
| *Macrotermes gilvus* | grass stalks, leaves, grass blades, fallen mango leaves | [23] |
| *Macrotermes gilvus* | wood, bark and grass | [24] |
| *Macrotermes gilvus* | wood | [25] |
| *Macrotermes natalensis* | grass, leaves, thorns, fruit husks, twiglets | [26] |
| *Macrotermes natalensis* | dry wood, cow dung, decaying wood and bark | [27] |
| *Macrotermes natalensis* | live wood | [20] |
| *Macrotermes natalensis* | wood litter | [28] |
| *Macrotermes falciger* | live wood | [2] |
| *Macrotermes falciger* | live wood | [20] |

| | | |
|---|---|---|
| *Macrotermes falciger* | dung, wood and grass litter | [15] |
| *Macrotermes malaccensis* | live wood and roots | [29] |
| *Macrotermes ukuzii* | dung, wood and grass litter | [15] |
| *Microtermes* sp. | wood and grass | [3] |
| *Microtermes* sp. | wood (dead and fresh material) | [30] |
| *Microtermes* sp. | dung and wood | [6] |
| *Microtermes* sp. | roots, grass and wood litter | [4] |
| *Microtermes* sp. | dead wood | [14] |
| *Microtermes* sp. | dead plant material | [5] |
| *Microtermes* spp. | log and twigs | [18] |
| *Microtermes* spp. | Fresh roots and dry wood | [2] |
| *Microtermes* spp. | small twigs, broken branches, woody, grass, and leaf litter, | [11] |
| *Microtermes* spp. | leaves | [17] |
| *Microtermes ?albopartitus* | wood | [1] |
| *Microtermes ?alluaudanus* | wood | [1] |
| *Microtermes ?aluco* | wood | [1] |
| *Microtermes alluaudanus* | dung, wood fragments | [31] |
| *Microtermes congoensis* | wood | [1] |
| *Microtermes depauperata* | wood | [1] |
| *Microtermes grassei* | wood | [1] |
| *Microtermes mycophagus* | wood, fresh plant material (trees and leaves) | [32] |
| *Microtermes obesi* | fresh leaves and wood | [33] |
| *Microtermes pakistanicus* | dead plant material, palm trees, sugar cane | [34] |
| *Microtermes subhyalinus* | sugar cane | [35] |
| *Microtermes toumodiensis* | wood litter | [36] |
| *Microtermes unicolor* | fresh leaves and wood | [37] |
| *Microtermes yemenensis* | fresh plant material | [38] |
| *Odontotermes*sp. | dung and wood | [6] |
| *Odontotermes* sp. | dead plant material | [5] |
| *Odontotermes* sp. | woody litter | [11] |
| *Odontotermes* sp. | dry wood, cow dung | [27] |
| *Odontotermes* sp. | wood, grass and leaf litter | [4] |
| *Odontotermes* sp. | dry wood | [2] |
| *Odontotermes* sp.1 | wood | [1] |
| *Odontotermes* sp.2 | wood | [1] |
| *Odontotermes* sp. near *badius* | dry wood | [2] |
| *Odontotermes sp.* near *kinarensis* | live roots | [2] |
| *Odontotermes* spp. | log and twigs | [18] |
| *Odontotermes* spp.1 | grass litter | [8] |
| *Odontotermes* spp. 2 | grass litter | [8] |
| *Odontotermes* sp. A | dead wood fragments from trees or logs | [17] |
| *Odontotermes* sp. A | dead wood | [14] |
| *Odontotermes* sp. A | wood and grass | [3] |
| *Odontotermes* sp. B | dead wood fragments from trees or logs | [17] |

| | | |
|---|---|---|
| *Odontotermes* sp. B | wood and grass | [3] |
| *Odontotermes* sp. B | dead wood | [14] |
| *Odontotermes* sp. C | dead wood fragments from trees or logs | [17] |
| *Odontotermes pauperans* | dead wood fragments from trees or logs | [17] |
| *Odontotermes pauperans* | monocot litter | [36] |
| *Odontotermes formosanus* | log and fresh leaves | [39] |
| *Odontotermes formosanus* | Dead wood | [40] |
| *Odontotermes badius* | dry wood, cow dung, | [27] |
| *Odontotermes badius* | dead wood, sound wood and live wood from eucalyptus | [2] |
| *Odontotermes badius* | dung, wood and grass litter | [15] |
| *Odontotermes boanicus* | dung and wood | [41] |
| *Odontotermes mediocris* | dung, wood fragments | [31] |
| *Odontotermes zambesiensis* | dung, wood fragments | [31] |
| *Odontotermes lokanandi* | fresh leaves and wood | [37] |
| *Odontotermes lokanandi* | fresh leaves and wood | [33] |
| *Odontotermes latericius* | dung and wood | [15] |
| *Odontotermes obesus* | roots | [42] |
| *Odontotermes nilensis* | millet canes and Acacia litter, cow dung | [19] |
| *Odontotermes transvaalensis* | live roots and dry wood | [2] |
| *Odontotermes transvaalensis* | dung and wood | [15] |
| *Odontotermes patruus* | dung and wood | [41] |
| *Odontotermes amaniensis* | wood | [43] |
| *Allodontermes* sp. | woody litter | [11] |
| *Allodontermes* sp. | wood and grass | [3] |
| *Allodontermes* sp. | dead wood | [14] |
| *Allodontermes* sp. | dung, wood and grass litter | [6] |
| *Allodontermes rhodesiensis* | sound wood (dry wood) of eucalyptus (dry) | [2] |
| *Allodontermes schulzei* | live and dead wood | [2] |
| *Synacanthotermes zanzibarensis* | wood fragments | [31] |
| *Synacanthotermes zanzibarensis* | Fresh roots and live wood | [2] |
| *Synacanthotermes heterodon* | wood | [1] |
| *Ancistrotermes* sp. | wood (dead and fresh material) | [30] |
| *Ancistrotermes* sp. | roots, grass and wood litter | [4] |
| *Ancistrotermes cavithorax* | log and twigs | [44] |
| *Ancistrotermes cavithorax* | wood litter | [36] |
| *Ancistrotermes crucifer* | dead wood fragments from trees or logs | [17] |
| *Ancistrotermes guineensis* | millet canes, wood | [19] |
| *Ancistrotermes latinotus* | dead wood | [14] |
| *Ancistrotermes latinotus* | live roots | [15] |
| *Ancistrotermes latinotus* | dung, wood and grass litter | [15] |
| *Protermes* sp. | dry wood (eucalyptus) | [2] |
| *Protermes birticeps* | dead wood fragments from trees or logs | [17] |
| *Protermes minutus* | leaves and dead wood fragments from trees or logs | [17] |
| *Protermes prorepens* | wood and litter | [1] |
| *Pseudacanthotermes militaris* | dead wood fragments from trees or logs | [17] |
| *Pseudacanthotermes militaris* | dung, wood and grass litter | [45] |
| *Pseudacanthotermes militaris* | dead leaves, twigs, branches, logs, sound wood. Live roots and live wood | [2] |

| | | |
|---|---|---|
| *Pseudacanthotermes militaris* | sugar cane | [46] |
| *Pseudacanthotermes militaris* | corn | [17] |
| *Pseudacanthotermes militaris* | live wood (tea) | [47] |
| *Pseudacanthotermes militaris* | rice | [48] |
| *Pseudacanthotermes militaris* | live roots (cassava) | [49] |
| *Pseudacanthotermes militaris* | sugar cane | [50] |
| *Pseudacanthotermes militaris* | wood and litter | [1] |
| *Pseudacanthotermes spiniger* | wood and litter | [1] |
| *Pseudacanthotermes spiniger* | dead leaves, twigs, branches, logs, sound wood, live wood | [2] |
| *Pseudacanthotermes spiniger* | wood and bark | [26] |
| *Pseudacanthotermes* spp. | grass litter | [8] |
| *Pseudacanthotermes* sp. | dead plant material | [5] |
| *Acanthotermes acanthorax* | wood and bark | [26] |
| *Acanthotermes* spp. | grass, live root, live wood, fresh leaves | [51] |
| *Acanthotermes acanthothorax* | wood and litter | [1] |

## Supplementary References


1. Eggleton, P.; Bignell, D. E.; Sands, W. A.; Waite, B.; Wood, T. G.; Lawton, J. H. The species richness of termites (Isoptera) under differing levels of forest disturbance in the Mbalmayo Forest Reserve, southern Cameroon. *J. Trop. Ecol.* **1995**, *11*, 85–98.

2. Nkunika, P. O. Y. A survey of the termite species associated with *Eucalyptus* plantations in Zambia. *Zambia J. Sci. Technol.* **1980**, *5*, 33–38.

3. Schuurman, G. Foraging and distribution patterns in a termite assemblage dominated by fungus-growing species in semi-arid northern Botswana. *J. Trop. Ecol.* **2006**, *22*, 277–287, doi:10.1017/S0266467405003044.

4. Longhurst, C.; Howse, P. E. The use of kairomones by *Megaponera foetens* (Fab.) (Hymenoptera: Formicidae) in the detection of its termite prey. *Anim. Behav.* **1978**, 2013–2018, doi:10.1016/0003-3472(78)90111-2.

5. Abe, T. Termite community in the grassland of Kenya with special reference to their feeding habits. In *Social insects and the environment. Proceedings of the 11th International Congress of IUSSI, 1990 (International Union for the Study of Social Insects).*; 1990; pp. 207–208.

6. Coaton, W.; Sheasby, J. Preliminary report on a survey of the termites (Isoptera) of South West Africa. *Cimbebasia Mem.* **1972**, *2*, 1–129.

7. Ouédraogo, E.; Mando, A.; Brussaard, L. Soil macrofaunal-mediated organic resource disappearance in semi-arid West Africa. *Appl. Soil Ecol.* **2004**, *27*, 259–267, doi:10.1016/j.apsoil.2004.03.003.

8. Mugerwa, S.; Nyangito, M.; Mpairwe, D.; Nderitu, J. Effect of biotic and abiotic factors on composition and foraging intensity of subterranean termites. *African J. Environ. Sci. Technol.*

**2011**, *5*, 579–588, doi:10.5897/AJEST11.088.

9. Lepage, M. G. L'impact des populations récoltantes de *Macrotermes michaelseni* (Sjostedt) (Isoptera, Macrotermitinae) dans un écosystème semi-aride (Kajiado-Kenya) - I - L'activité de récolte et son déterminisme. *Insectes Soc.* **1981**, *28*, 297–308, doi:10.1007/BF02223630.

10. Lepage, M. Foraging and food consumption of *Macrotermes subhyalinus*. In *VIIIth International Congress IUSSI*; 1977; pp. 249–252.

11. Dangerfield, J. M.; Schuurman, G. Foraging by fungus-growing termites ( Isoptera : Termitidae, Macrotermitinae ) in the Okavango Delta, Botswana. *J. Trop. Ecol.* **2000**, *16*, 717–731.

12. Boutton, T. W.; Arshad, M. A.; Tieszen, L. L. Stable isotope analysis of termite food habits in East African grasslands. *Oecologia* **1983**, *59*, 1–6, doi:10.1007/BF00388065.

13. Lepage, M. L′ impacte des populations récoltantes des *Macrotermes michaelseni* (Sjostedt) (Isoptera: Macrotermitinae) dans un écosystème semi-aride (Kaijaido – Kenya) II. Nourriture récoltée, comparison avec les grands herbivores. *Isectes Sociaux* **1981**, *28*, 309–319.

14. Schuurman, G. Decomposition rates and termite assemblage composition in semiarid Africa. *Ecology* **2005**, *86*, 1236–1249, doi:10.1890/03-0570.

15. Mitchell, B. L. Report on a survey of the termites of Zimbabwe. *Occas. Pap. Natl. Museum South. Rhod.* **1980**, *6*, 187 – 323.

16. Lepage, M. *The impact of foraging populations of the termite Macrotermes subhyalinus on a semi-arid ecosystem (Kajiado, Kenya).*; Nairobi, 1978.

17. Wood, A. T. G.; Johnson, R. A.; Bacchus, S.; Shittu, M. O.; Anderson, J. M. Abundance and Distribution of Termites ( Isoptera ) in a Riparian Forest in the Southern Guinea Savanna Vegetation Zone of Nigeria. *Assoc. Trop. Biol. Conserv.* **1982**, *14*, 25–39.

18. Collins, N. M. The role of termites in the decomposition of wood and leaf litter in the Southern Guinea savanna of Nigeria. *Oecologia* **1981**, *51*, 389–399, doi:10.1007/BF00540911.

19. Rouland, C.; Lepage, M.; Chotte, J. L.; Diouf, M.; Ndiaye, D.; Ndiaye, S.; Seugé, C.; Brauman, A. Experimental manipulation of termites (Isoptera, Macrotermitinae) foraging patterns in a Sahelo-Sudanese savanna: Effect of litter quality. *Insectes Soc.* **2003**, *50*, 309–316, doi:10.1007/s00040-003-0680-6.

20. Sands, W. A. Observations on termites destructive to trees and crops. *North. Reg. Min. Agric. Samaru Res. Bull.* **1962**, *26*, 1–14.

21. Korb, J.; Linsenmair, K. E. Evaluation of predation risk in the collectively foraging termite *Macrotermes bellicosus*. *Insectes Soc.* **2002**, *49*, 264–269, doi:10.1007/s00040-002-8312-0.

22. Johjima, T.; Inoue, T.; Ohkuma, M.; Noparatnaraporn, N.; Kudo, T. Chemical Analysis of Food Processing by the Fungus-Growing Termite *Macrotermes gilvus*. *Sociobiology* **2003**, *42*,

815–824.

23. Bathellier, J. Contribution à l'etude systematique et biologique des Termites de l'Indochine. *Faune Colon. Fran* **1927**, *1*, 125–365.

24. Kalshoven, L. G. E. Observations on *Macrotermes gilvus* Holmgr. In Java-3 Accumulations of finely cut vegetable matter in the nests. *Insectes Soc.* **1956**, *3*, 455–461, doi:10.1007/BF02225765.

25. Acda, M. N. Foraging Populations and Territories of the Tropical Subterranean Termite *Macrotermes gilvus* (Isoptera: Macrotermitinae). *Sociobiology* **2004**, *43*, 169–177.

26. Grassé, P. P.; Noirot, C. La transmission des flagellés symbiotiques et les aliments des termites. *Bull. Biol. Fr. Belg.* **1945**, *79*, 273–292.

27. da Costa, R. R.; Hu, H.; Pilgaard, B.; Sabine, S. M.; Schückel, J.; Pedersen, K. S. K.; Kračun, S. K.; Busk, P. K.; Harholt, J.; Sapountzis, P.; Lange, L.; Aanen, D. K.; Poulsen, M. Enzyme activities at different stages of plant biomass decomposition in three species of fungusgrowing termites. *Appl. Environ. Microbiol.* **2018**, *84*, e01815-17, doi:10.1128/AEM.01815-17.

28. Meyer, V. W.; Crewe, R. M.; Braack, L. E. O. Estimates of food consumption by the fungus-growing termite *Macrotermes natalensis* in a South African savanna-woodland. *S. Afr. J. Sci.* **2003**, *99*, 207–208.

29. Santoso, E.; T., H. Pest and disease control of timber estates in East Kalimantan. *J. For. Res. Dev.* **1991**, *7*, 14–17.

30. N'Dri, A. B.; Gignoux, J.; Konaté, S. Food preferences and foraging strategies of wood-feeding termites in a West African savanna. *Curr. Sci.* **2018**, *114*, 186–192, doi:10.18520/cs/v114/i01/186-192.

31. Buxton, R. D. Termites and the turnover of dead wood in an arid tropical environment. *Oecologia* **1981**, *51*, 379–384.

32. Iqbal, N.; Saeed, S.; Evans, T. A.; Kwon, Y. J. Foraging activity and population estimation of *Microtermes mycophagus* Desneux (Isoptera: Termitidae: Macrotermitinae) in Multan, Punjab, Pakistan. *Entomol. Res.* **2015**, *45*, 51–57, doi:10.1111/1748-5967.12094.

33. Sattar, A.; Naeem, M.; ul-Haq, E. Impact of environmental factors on the population dynamics, density and foraging activities of *Odontotermes lokanandi* and *Microtermes obesi* in Islamabad. *Springerplus* **2013**, *2*, 1–7, doi:10.1186/2193-1801-2-349.

34. Lee, C. Y.; Ngee, P. S.; Lee, L. C. Foraging populations and territories of a mound-building subterranean termite, *Microtermes pakistanicus* (Isoptera: Macrotermitinae). *Sociobiology* **2003**, *41*, 307–316.

35. Mora, P.; Rouland, C.; Renoux, J. Foraging, nesting and damage caused by *Microtermes subhyalinus* (Isoptera: Termitidae) in a sugarcane plantation in the Central African Republic.

*Bull. Entomol. Res.* **1996**, *86*, 387, doi:10.1017/S0007485300034970.

36. Jouquet, P.; Boulain, N.; Gignoux, J.; Lepage, M. Association between subterranean termites and grasses in a West African savanna: Spatial pattern analysis shows a significant role for *Odontotermes* n. *pauperans*. *Appl. Soil Ecol.* **2004**, *27*, 99–107, doi:10.1016/j.apsoil.2004.05.002.

37. Sattar, A.; Misbah, M.; Salihah, Z.; Khatoon, R. Foraging Activity of *Microtermes unicolor* Synder and *Odontotermes lokanandi* Chatterjee and Thakur ( Termitidae, Isoptera ) in Peshawar, Pakistan. *Suranaree J. Sci. Technol.* **2007**, *15*, 69–74.

38. Al-Hemyari, A. A. Factors affecting foraging activity of *Microtermes yemenensis* (Isoptera: Termitidae). *Ann. Agric. Sci.* **1994**, *32*, 987–995.

39. Soleymaninejadian, E.; Ji, B. Z.; Liu, S. W.; Yang, J. J.; Wang, H. J.; Ding, F. Polyethism in *Odontotermes formosanus* Shiraki. *Int. J. Biol. Med. Res.* **2014**, *5*, 4231–4238.

40. Chiu, C. I.; Yeh, H. T.; Li, P. L.; Kuo, C. Y.; Tsai, M. J.; Li, H. F. Foraging Phenology of the Fungus-Growing Termite *Odontotermes formosanus* (Blattodea: Termitidae). *Environ. Entomol.* **2018**, *47*, 1509–1516, doi:10.1093/ee/nvy140.

41. Kemp, P. B. The termites of north-eastern Tanganyika: their distribution and biology. *Bull. Entomol. Res.* **1955**, *46*, 112 – 135.

42. Malik, M. U.; Javed, H.; Ayyaz, M. Evaluation of Different Groundnut *Arachis hypogea* L. Cultivars Against Termites, *Odontotermes obesus* ( Rambur ) in Rawalpindi, Pakistan. *Turkish J. Agric. - Food Sci. Technol.* **2015**, *3*, 448–452.

43. Burchard, I. On the removal of herbivore dung by *Odontotermes* spp. in Africa. *Sociobiology* **1989**, *15*, 261.

44. Collins, N. M. The role of termites in the decomposition of wood and leaf litter in the Southern Guinea savanna of Nigeria. *Oecologia* **1981**, *51*, 389–399, doi:10.1007/BF00540911.

45. Mitter, C.; Farrell, B.; Wiegmann, B. The Phylogenetic Study of Adaptive Zones: Has Phytophagy Promoted Insect Diversification? *Am. Nat.* **1988**, *132*, 107–128, doi:10.1086/284840.

46. Wheatley, P. E.; Crowe, T. J. *Pest Handbook: The Recognition and Control of the More Important Pests of Agriculture in Kenya*; Government Printer: Nairobi, Kenya, 1967;

47. Benjamen, D. M. Insects and mites on tea in Africa and adjacent islands. *East Africa Agric. For. J.* **1968**, *33*, 345–357.

48. Togola, A.; Kotoklo, E. A.; Nwilene, F. E.; Amevoin, K.; Glitho, I. A.; Oyetunji, O. E.; Kiepe, P. Specific diversity and damage of termites on upland rice in Benin. *J. Entomol.* **2012**, *9*, 352–360, doi:10.3923/je.2012.352.360.

49. Sands, W. A. Termites as pests of tropical food crops. *Pest Artic. News Summ.* **1973**, *19*, 167–177.

50. Dibangou, V.; Mora, P.; Celini, L.; Rouland-LeFèvre, C. Spatial distribution and density of fungus-growing termite *Pseudacanthotermes militaris* (Isoptera: Macrotermitinae) in the Congo Republic. *Asian J. Biol. Sci.* **2012**, *5*, 406–416.

51. Nair, K. S. S. *Tropical forest insect pests: Ecology, impact, and management.*; Cambridge University Press, 2007.
